# Deep Learning Multi-Modal Melanoma Detection: Algorithm Development and Validation

**DOI:** 10.2196/66561

**Published:** 2025-08-13

**Authors:** Nithika Vivek, Karthik Ramesh

**Affiliations:** 1 Del Norte High School, 16601 Nighthawk Ln, San Diego, CA, 92127, United States, 1 619 458 5059; 2 Department of Internal Medicine, University of California, Los Angeles, Los Angeles, CA, United States

**Keywords:** melanoma, dermatology, artificial intelligence, deep learning, multi modal, geriatric, metastasis, seborrheic keratosis, patient image data, accuracy, computer vision

## Abstract

**Background:**

The visual similarity of melanoma and seborrheic keratosis has made it difficult for older patients with disabilities to know when to seek medical attention, contributing to the metastasis of melanoma.

**Objective:**

This study aimed to present a novel multimodal deep learning-based technique to distinguish between melanoma and seborrheic keratosis.

**Methods:**

Our strategy is three-fold: (1) use patient image data to train and test three deep learning models using transfer learning (ResNet50, InceptionV3, and VGG16) and one author-designed model, (2) use patient metadata to train and test a deep learning model, and (3) combine the predictions of the image model with the best accuracy and the metadata model, using nonlinear least squares regression to specify ideal weights to each model for a combined prediction.

**Results:**

The accuracy of the combined model was 88% (195/221 classified correctly) on test data from the HAM10000 dataset. Model reliability was assessed by visualizing the output activation map of each model and comparing the diagnosis patterns to that of dermatologists. The addition of metadata to the image dataset was key to reducing the false-negative and false-positive rates simultaneously, thereby producing better metrics and improving overall model accuracy.

**Conclusions:**

Results from this experiment could be used to eliminate late diagnosis of melanoma via easy access to an app. Future experiments can use text data (subjective data pertaining to how the patient felt over a certain period of time) to allow this model to reflect the real hospital setting to a greater extent.

## Introduction

Incidence rates of melanoma have been on an increase since 1999, with 15.1 per 100,000 in 1999 and rising to 23.0 per 100,000 in 2021 [1]. In contrast, seborrheic keratosis is a benign skin appearance that commonly occurs in older adults. While the pathology, epidemiology, and histology of melanoma and seborrheic keratosis are well understood [2, 3, 4, 5], on a surface level, these 2 lesions can seem almost identical to the untrained eye, making it difficult for individuals to know when to seek care [6]. Delayed care can allow a malignant lesion to progress into metastatic melanoma. As the stage of melanoma progresses, the survival rate can decrease as much as 67% [7]. Thus, timely diagnosis and treatment are paramount.

The current diagnostic paradigm has not significantly advanced despite staggering technological leaps. A typical process involves a patient visiting a primary care clinic, followed by a referral to a dermatologist if there are any unusual skin lesions [8]. The dermatologist repeats the skin exam, and then further performs biopsies or excision as required [9]. These samples are sent for pathology, which makes the final diagnosis. This process requires an iterative process involving appropriate presentation to a primary care provider, appropriate referral, appropriate visual analysis, appropriate surgical excision, all before a diagnosis can be made [10].

Deep learning models have commonly been used to encourage at-home, self-diagnosis, or easier physician diagnosis of melanoma [11, 12]. One such experiment used an Iterative Dichotomiser 3 (ID3) algorithm to learn rules from image data using texture patterns, a method known as automatic induction [13]. Another method employed transfer learning [14] and used ResNet152 to develop a binary classifier between benign and malignant skin lesions [15].

Table 1 describes the average area under the curves (AUCs) for medical imaging segmentation for various dermatological machine learning models proposed in literature. The best-performing model was a combination of ResNet-50 and InceptionV3, with an accuracy of 80%. Most of these approaches aim to optimize models through transfer learning and various preprocessing techniques in an attempt to increase accuracy.

**Table 1.** Comparison of machine learning approaches taken in 65 dermatological applications across the internet, with analysis on either the HAM10000 dataset, ISIC dataset, DFUC dataset, or other common datasets ranging from 46‐33126 data points for evaluation [14].

Model	Accuracy (area under the curve)
ResNet-50	71.620
VGG	68.408
InceptionV3	74.311
ResNet-50 and Inception V3	85.977
ResNet-50 and VGG	83.065
ID3^a^	71.000
BottleNeckCSP	81.000

a ID3: Iterative Dichotomiser 3.

Tabular data has been extensively used in various health applications, serving as the basis of many prediction algorithms and machine learning models [16]. One relevant dermatological example used clinical features to represent the redness, flakiness, definite border extent, and other qualities to classify 6 types of erythemato-squamous skin diseases using the UCI Dermatology dataset [17]. Past tabular metadata for health applications have been used to diagnose other, nondermatological-related diseases. A Dual Bayesian ResNet50 model was used to train metadata regarding heart murmurs using XGBoost [18]. Broader applications of tabular metadata have been used through a method called MediTab, in which diverse, out-of-sample data is consolidated and aligned to improve prediction accuracy [19]. Time progression tabular deep learning was used for hypercholesterolemia, in which a multistage deep learning architecture was used to analyze familial hypercholesterolemia [20]. However, this method was not integrated with image data and was purely reliant on tabular data.

Image and tabular predictions can be combined into a hybrid model by using nonlinear least squares regression (NLS) by incorporating both image and tabular predictions in a unified regression model. Past studies have found NLS useful for fusion of heterogeneous sources of data due to its ability to model complex, nonlinear relationships inherent in such data [21]. NLS is a common technique used to fit a model to data by minimizing the square sum of residuals or the squared differences between observed data points and values predicted by the nonlinear model. Minimizing this difference allows for the predictions to more accurately reflect the true value. NLS has been used in pharmacokinetics to understand drug absorption, distribution, metabolization, and excretion [22]. Other applications of NLS appear in tumor growth analysis and medical imaging to enhance image quality [23,24].

Because melanoma prevalence can vary among different demographics, image inputs or metadata inputs alone may not be sufficient in formulating an accurate diagnosis [25]. This paper aims to build on the previous experiments stated and incorporate metadata into the model inputs. While NLS regression has been previously commonly used on raw medical data, this application of NLS leverages its square residuals minimizing abilities to determine ideal weights for the combination of tabular and image data at the output. Finally, providing the model with multiple input modalities helps capture heterogeneous factors that decrease the chances of the model formulating false patterns during classification.

## Methods

### Overview

Our multimodal deep learning architecture assembly is threefold: (1) use patient image data to train and test three deep learning models using transfer learning (ResNet50, InceptionV3, and VGG16) and one author-designed model, (2) use patient metadata to train and test a deep learning model, and (3) combine the predictions of the image model with the best accuracy and the metadata model, using nonlinear least squares regression to specify ideal weights to each model for a combined prediction.

### Dataset Analysis

The data used in this experiment was obtained from the HAM10000 dataset [26]. 2259 images were taken from the practice of Cliff Rosendahl consecutively starting 2008 until 2017. 7756 images were taken from the University of Vienna in 1988. Because images were collected from different time periods, some were preprocessed with enhanced contrast and zoom while others were not. While all types of skin conditions were captured in the dataset, for the purposes of this analysis, those images not classed as seborrheic keratosis or melanoma were removed. There were a total of 2210 images, with 50% (1105 images) belonging to melanoma and 50% (1105 images) belonging to seborrheic keratosis.

### Data Preparation and Cleaning

Deduplication based on lesion ID was performed to prevent train and test overlap due to the presence of preaugmented images. Using the Python package TensorFlow, the data was split into train (70% or 1547/2210 images), test (10% or 221/2210 images), and validation (20% or 442/2210 images) and then into batches to allow for parallel processing. All splits of data were then augmented and normalized to reduce overfitting and ensure equal scaling of pixel values.

### Build and Train Image Models

**Figure 1.**
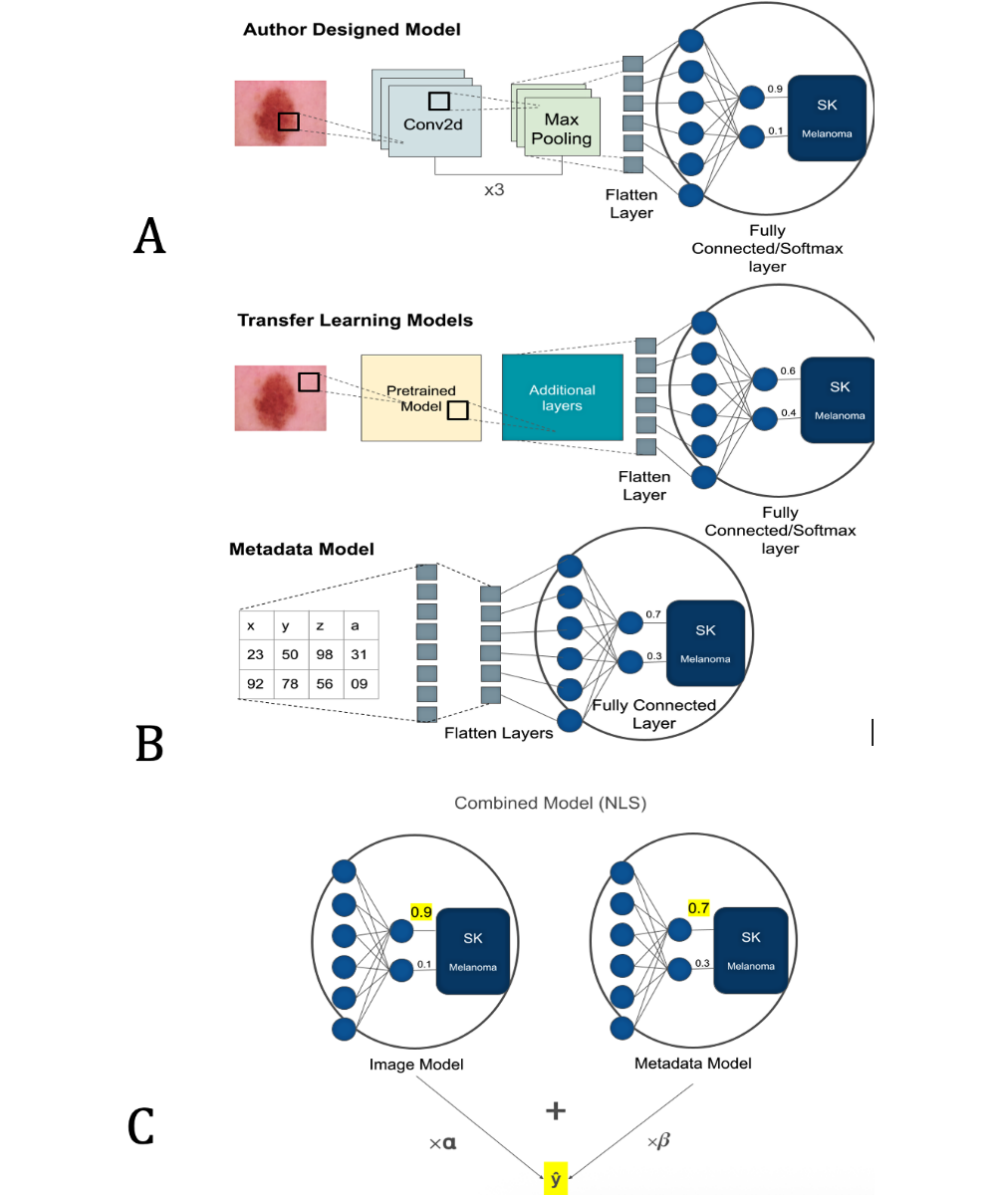
Architecture of the author designed and transfer learning models. (A) describes the architecture of the image model with three convolutional layers and transfer learning layers, (B) describes the metadata model for processing structured data, and (C) outlines the NLS method used to combine the predictions from each model.

Four image models were developed as depicted in Figure 1: an author-designed model and 3 transfer learning models. The author-designed model contained 3 convolutional layers with max pooling layers following each one, one flatten, and 2 dense layers. Convolutional layers help with extracting features from the image by applying certain weights to them, and max pooling layers assist in this by performing dimensionality reduction on the convolution layer output. Flatten layers once again change the dimensions, and dense layers help with forming global connections between the learned input. The output of this model was determined by the SoftMax layer, which generates a probability of the input belonging to the malignant class. The transfer learning models include pretrained ResNet50, InceptionV3, and VGG16, which were frozen to keep existing memory, and additional trainable layers were added to fine-tune the overall system. Dropouts of 0.3 and L2 weights of 0.01 were used to attempt to mitigate overfitting. All models were run for the same number of epochs, and the run time per epoch was recorded. A larger time was spent on training the transfer learning models because they have more convolutional layers and therefore take longer to output a feature map from each layer.

### Improving Image Model Accuracy

**Figure 2.**
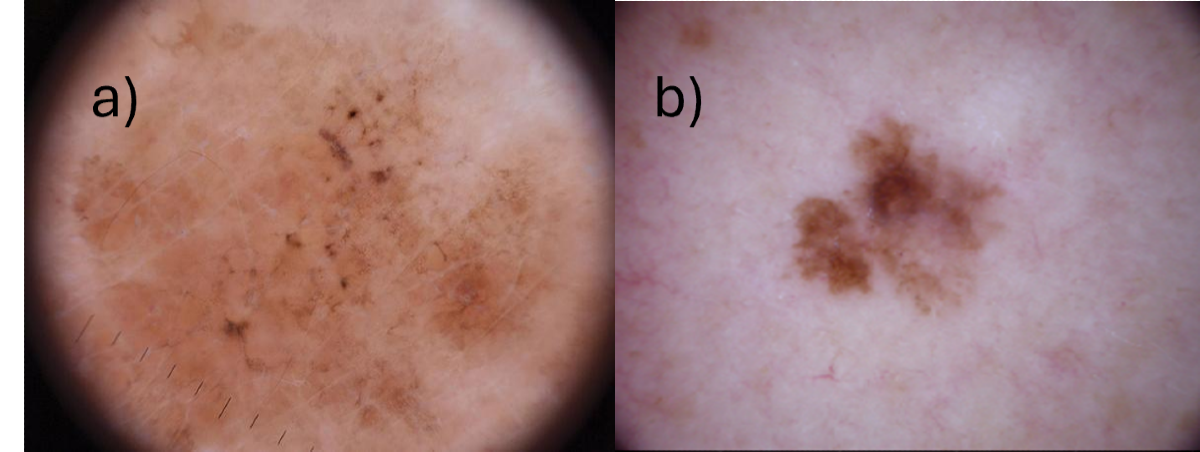
Examples of faulty and good images. Specific metrics were used for data cleaning. (A) Faulty images, with dots per inch (DPI) <72 and approximate zoom <600×800px were removed. (B) Good images, with DP>72 and approximate zoom 600 × 800px were kept. 200 out of 2400 faulty images were removed from the dataset using these specifications, 100 from each class.

To improve model accuracy, further data cleaning was performed. Train, test, and validation datasets were manually parsed through with the following metrics in mind:<72 DPI and <600 x 800 px with visuals depicted in Figure 2. 8.3% (183/2210) of the data was eliminated this way and rerun with the same model structure to analyze the effect of image quality on model accuracy.

### Metadata Cleaning and Run

After optimizing and validating the image model, the metadata was cleaned and split similar to the image data. A train, test, and validation dataset was built that matched that of the images using matching image IDs to ensure controlled training. Categorical columns were made numerical through manual mapping, and the data was standardized using a built-in package called StandardScaler. A simple model architecture with only dense layers was used as visual patterns are not necessary for structured data. However, even without convolutional layers, global knowledge pattern formation was achieved through dense (fully connected) layers that connected each “node,” or learned pattern, to each other.

### Combining the Two: Non-Linear Least Squares (NLS) Regression

The image and metadata model output SoftMax probabilities for each class (melanoma and seborrheic keratosis). The NLS regression method was applied to determine optimal weights for combining each model’s prediction. The coefficients were determined through analysis of image and metadata outputs for the training dataset.


(1) $$0.75_{{\text{X}}_{1}}+0.25_{{\text{X}}_{2}}=\hat{y}$$

The above equation, outputted from the NLS function, describes the weights applied to both image (x_1_) and metadata (x_2_) model outputs to achieve an ideal accuracy. ŷ represents the combined prediction, with values>0.5 being classified as malignant (melanoma) and values<0.5 being classified as seborrheic keratosis.

### Ethical Considerations

No human participants were involved in this research. All data used in this research was obtained from the HAM10000 dataset, an open source and publicly available dataset. The authors of the HAM10000 dataset state that data sources were approved by the ethics committee at the Medical University of Vienna (Protocol No. 1804/2017) and the institutional ethics board at the University of Queensland (Protocol No. 2017001223).

## Results

### Comparing Model Accuracies

The simple model had the highest accuracy of 83.4% (369/442 images classified correctly) on validation data. All transfer learning models had high training accuracy but low validation accuracies, showing signs of overfitting. With the number of epochs in training constant, the transfer learning models show significantly more training time than the self-built model, as well as depicted in Table 2.

**Table 2.** Comparing model accuracies.

Model name	Training accuracy, N=1547, n (%)	Validation accuracy, N=442, n (%)	Number of epochs	Run time per epoch
ResNet50	1526 (98.65)	240 (54.29)	500	229 seconds
InceptionV3	1512 (97.75)	296 (67.04)	500	315 seconds
VGG16	1524 (98.52)	270 (61.13)	500	401 seconds
Self-Built Model (pre-data cleaning)	1242 (80.27)	348 (78.62)	500	2 seconds
Self-Built Model (post-data cleaning)	1488 (96.2)	369 (83.4)	500	2 seconds

### ROC Curves

ROC (receiver operating characteristic) curves were plotted as another method of showcasing the accuracy of each model. The self-built model had the highest AUC of 83% (369/442 images classified correctly) on validation data, consistent with the self-built model accuracy from the validation data. This model reaches its highest true-positive rate while achieving lower false-positive rates than the transfer learning models. The transfer learning models had significantly lower AUCs with ResNet50 approaching the random guess line.

### Validating Image Model

Saliency maps on test data illustrate the region of interest identified by different convolutional neural network architectures, allowing for greater model reliability and interpretability. They were generated from the last convolution layer, to help visualize which regions of an image are important for final classification. Each model demonstrates varying focus patterns, reflecting differences in feature extraction and attention and accounting for varying accuracies across all models.

### Combined Model: Confusion Matrices

The classification performance of the image, metadata, and combined models was evaluated through confusion matrices reflecting sensitivity and specificity. The image-based model shows a balanced distribution of correct classifications, achieving a true-negative rate of 42% (93/221) and a true-positive rate of 41% (91/221) on test data. The metadata-based model exhibited lower overall performance. When both image and metadata inputs were integrated, better performance was achieved across all metrics.

## Discussion

### Comparing Model Accuracies

Contrary to what was expected, the transfer learning models appear to perform worse than the author-designed model. The differences in model accuracy can be attributed to model architecture, particularly the number of convolutional layers. Transfer learning models have far more convolutional layers than the self-built model (ie, ResNet50 has 50 convolutional layers while the self-built model has only 3). As the number of convolutional layers increases, the ability of the model to detect more complex and finer features increases. Therefore, the transfer learning models are more susceptible to overfitting as they can detect more minute details like hair and wrinkles. This accounts for the overfitting occurring in the transfer learning models as seen in the large difference between training and validation accuracy.

ResNet50 differs from the author-constructed model as it contains a residual layer that directly connects the output layers to the input layers as opposed to “stacking” them. The author constructed model optimizes the accuracy by using backpropagation, where the gradients used to determine the minimum loss value are calculated using the chain rule. Rather than using the chain rule, ResNet avoids the subsequent derivation between each layer and instead connects each output to the input. While this is important for models with a large number of convolutional layers, the author constructed model only contains 3 convolutional layers, so the effect of chain rule is less amplified, deeming the residual layer unnecessary. Inception V3 differs from the author-constructed model as it uses parallel convolutional layers to analyze a wider feature range in the input images. However, because melanoma is often centered in one specific region and is attributed with a set of consistent features defined by the ABCDE rule, the detection of too many features is harmful. VGG16 specializes in using smaller kernel strides to center on more minute features, which can lead to overfitting in this situation as small details in the skin are not vital and sometimes confusing in making a classification. While past studies have shown that ResNet50 and InceptionV3 perform well in these applications, the ability of the simple model to generalize to this particular problem makes it better compared to these previous approaches.

**Figure 3.**
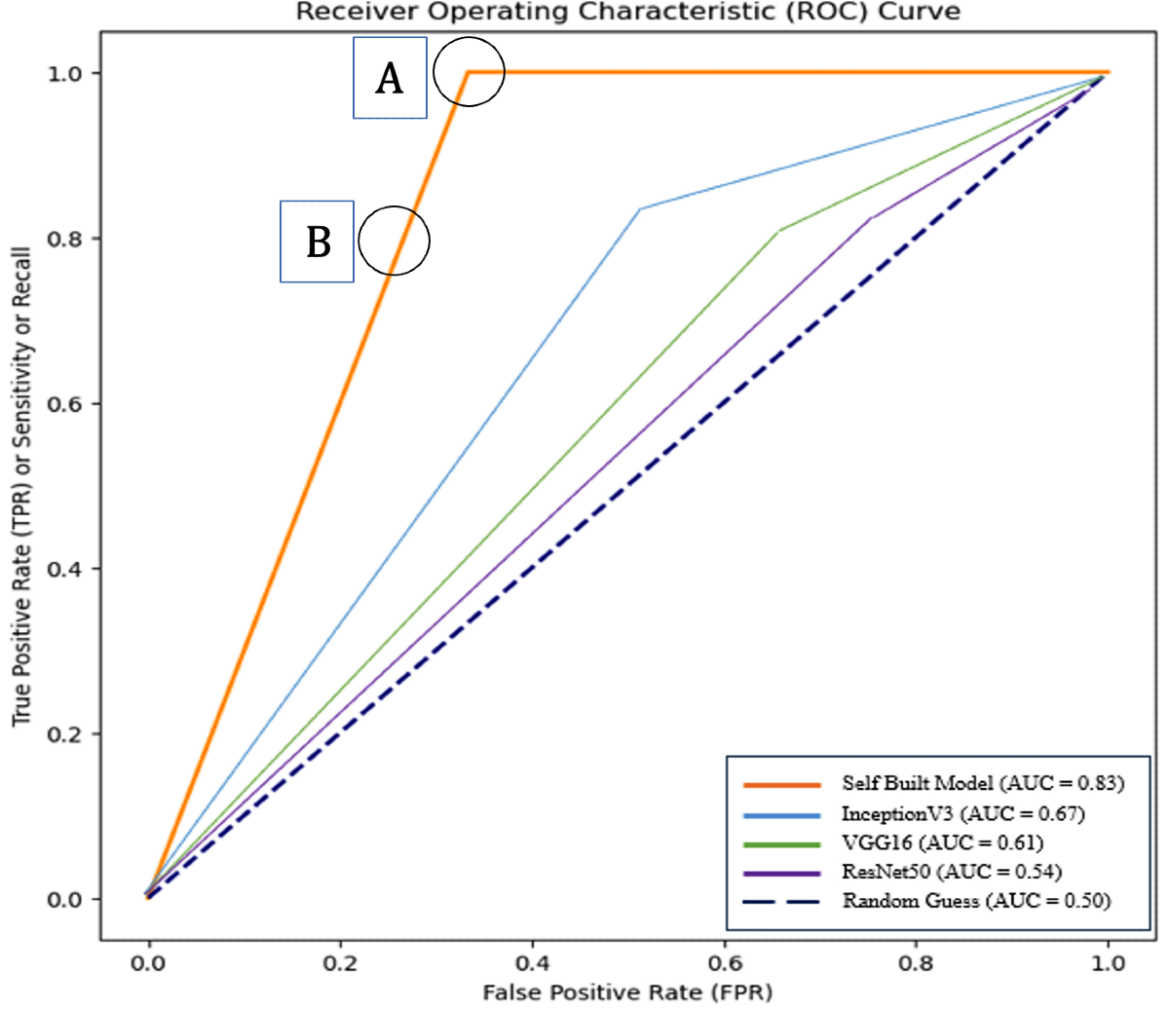
ROC curves: receiver operating characteristic curves plot the false-positive rate versus the true-positive rate. The ideal point on the curve is (0,1). FPR: false-positive rate; ROC: receiver operating characteristic; TPR: true-positive rate.

In real-world deployment, frontend image capture tools [27] will ensure image inputs conform to these predetermined metrics as shown in Figure 3, thereby increasing usability of the model. Upon deployment of this model to the primary care office, physicians are further advised to take good quality images of their patients’ lesions to ensure accurate diagnosis.

### ROC Curves

ROC curves in Figure 3 are used to determine a cutoff point that optimizes the sensitivity and specificity of a specific test [28]. In medical applications, this is especially important since false-negative results could be life-threatening. As the false-negative rate is a direct function of the true-positive rate, in order to lower the false-negative rate, the true-positive rate must be increased, even if it comes at the expense of the false-positive rate. Consequently, point A would be preferred to point B.

In addition, ROC curves can also be a measure of accuracy through the AUC depicted in the key shown in Figure 3. As the models get worse (as shown by the accuracies in Table 3), the ROC curve moves further away from the ideal point (0,1) and towards the random guess line [29].

**Table 3.** The final testing accuracy of the combined model is significantly higher than the existing accuracies from the literature review, which averaged around 70%.

Model type	Sensitivity	Specificity	Testing accuracy
Image	0.82	0.84	0.83
Metadata	0.76	0.52	0.64
Combined	0.875	0.875	0.875

### Heatmaps

**Figure 4.**
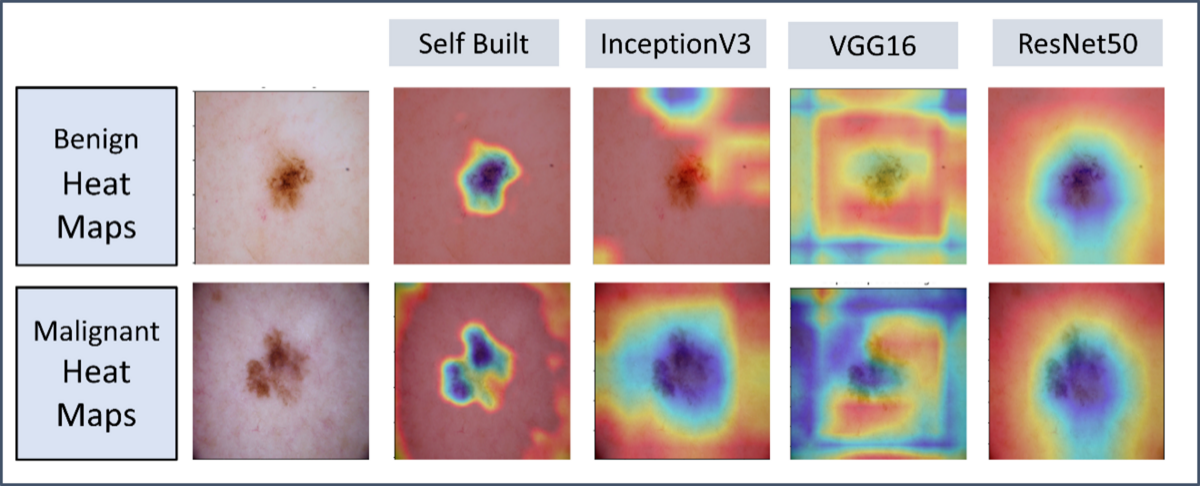
Comparison of Grad-CAM heat maps for benign and malignant skin lesions using different CNN architectures (Self-Built, InceptionV3, VGG16, ResNet50) for one example from each class.

A major problem in artificial intelligence models today is lack of interpretability [30]. Artificial intelligence is often referred to as a “black box” with limited explainability regarding its decisions [31]. However, Figure 4 allows users to “see through the eyes” of the model through heatmaps.

The author-designed model has a more “fixed” area of concentration as opposed to the other three transfer learning models. However, unlike InceptionV3, ResNet50 offers human interpretability and appears to follow the pattern presented in the author-designed model to a limited extent. However, it fails to capture differences between benign and malignant lesions as shown in the similar weight distributions between the 2 classes.

As shown in Figure 4, the author-designed model that performed the best appears to primarily look at the differences in border between the two lesions, connecting back to the ABCDE method used by dermatologists for clinical diagnosis [32]. This gives the model more reliability, as it is dissecting the image similar to how a dermatologist would.

### Confusion Matrices

**Figure 5.**
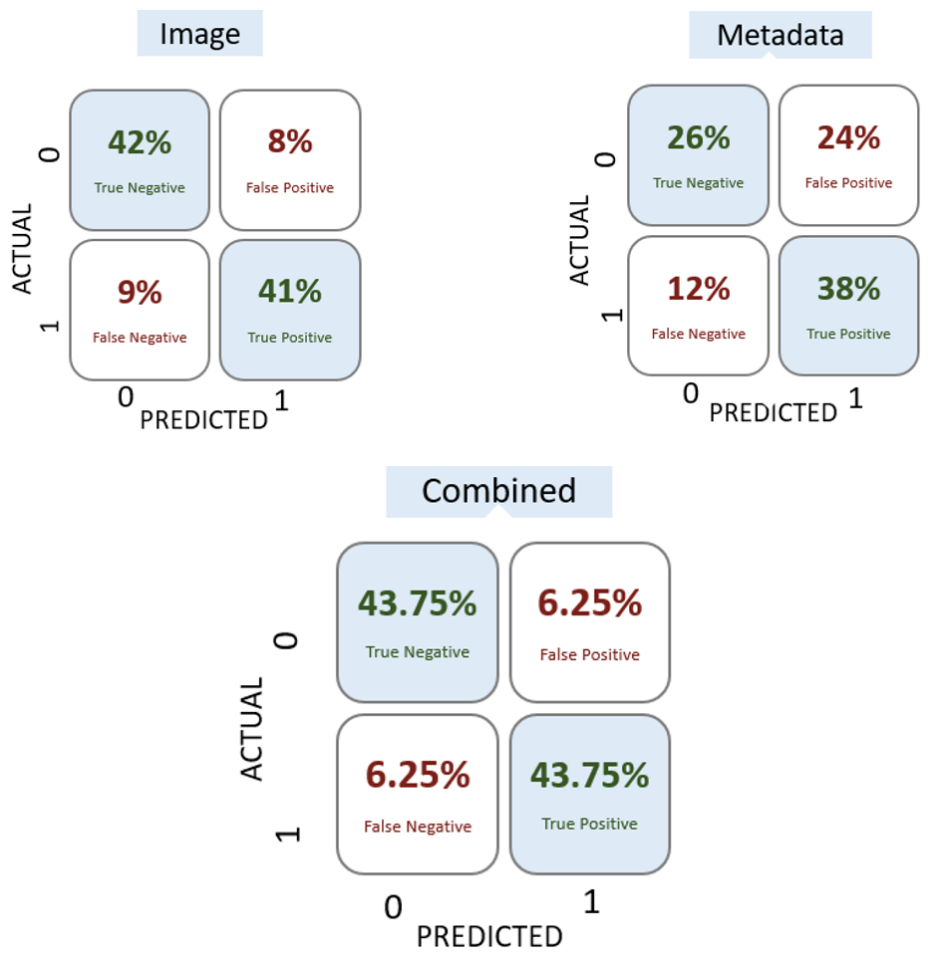
Confusion matrices depicting various metrics (false-positive and false-negative rates) as related to the ROC curve. A cutoff of 0.5 was used for prediction, with predictions over 0.5 being classified as melanoma. ROC: receiver operating characteristic.

Referring back to Figure 5, as the true-positive rate increases, it does so at the expense of the false-positive rate until a certain saturation point (A). Therefore, the 9% false-negative rate shown on the image confusion matrix (top left of Figure 5) can only be reduced at the expense of increasing the false-positive rate. The incorporation of the metadata adds critical heterogeneous information enabling the joint system to achieve a higher true-positive rate (lower false-negative rate) while simultaneously lowering the false-positive rate as shown in Figure 5 below. This thereby allows for the significant improvement in overall model accuracy as shown in Table 2.

### Comparison to Past Studies

Past work on dermatological applications of machine learning is compiled in Table 1, showing an accuracy ranging around 75%. The AUC of the transfer learning models (ResNet50, InceptionV3, and VGG16) matches that of past experiments. This study showcases an improvement of overall accuracy through the incorporation of additional metadata as well as constructing a simple model with fewer convolutional layers. These two approaches were successful in increasing the overall accuracy to 87.5% (194/221), showing promising implications for a multimodality approach to deep learning in dermatology.

### Applications and Improvements for Future Studies

Out of sample testing will be used through the deployment of this model in local hospital settings in cases with known diagnoses to ensure model feasibility and usability outside the controlled environment of HAM10000. To achieve this, this model will be employed in local dermatological centers and results will be compared against dermatologist-determined diagnosis to determine out-of-sample accuracy.

Cross-validation using different train-test-validation splits will be tested to increase the confidence of the model with access to more storage and compute units. To make this possible, a resource-efficient approach to training a convolutional neural network is necessary as images occupy a large amount of storage space.

Currently, the model does poorly when presented with patients aged 40 and younger as well as lesions present on curved areas of the body such as the eyelids. This is due to the lack of data from these demographics and areas, forcing the model to use generalized patterns to predict on these data points. Access to more granular metadata from younger patients and certain areas of the model can help address this issue. However, given the predominance of melanoma in older age groups, the authors believe this to be a natural obstacle of diagnosis in unusual populations.

As machine learning is a rapidly growing field, many new techniques can be used to improve the accuracy of the model. Combining metadata and image model predictions can be done through deep learning rather than regression, thereby enabling end-to-end joint training of the system to improve accuracy. Alternate architecture designs that combine image and metadata at the input or intermediate layers can also be explored. Additionally, using more granular metadata with less repetitions and more variations (eg, more data on different ages) can decrease the possibility of overfitting.

Using text data can also be a major change to this experiment. While this study only used structured data (patient metadata) and image data, in the real hospital setting, anecdotes, pain scale, lesion progression, and other descriptive factors can greatly influence a doctor when making a diagnostic decision. Using these records and combining them into the deep learning network through natural language processing can improve robustness and applicability of this model to the real world.

In order to make the application useful to a wider range of common citizens, making the model more robust by supporting a multi-way classification will allow older patients to use it in the home setting. Training the model on multiple types of lesions will motivate a more patient-friendly output as simply differentiating between benign and malignant eliminates the need to narrow down lesion possibilities.

### Conclusion

In this manuscript, we introduce a multimodal technique that employs heterogeneous forms of data to produce a probability of the lesion belonging to either class. The model expands upon current model architectures and is adapted and trained for the specific problem at hand. This strategy can be applied to a multitude of medical applications in addition to current studies to provide a more comprehensive diagnosis of a certain disease through the addition of multiple data modalities.

## Abbreviations

AUC: area under the curves
ID3: Iterative Dichotomiser 3
NLS: nonlinear least squares regression
ROC: receiver operating characteristic
